# Chromatin accessibility and cell cycle progression are controlled by the HDAC-associated Sin3B protein in murine hematopoietic stem cells

**DOI:** 10.1186/s13072-024-00526-w

**Published:** 2024-01-23

**Authors:** Alexander Calderon, Tamara Mestvirishvili, Francesco Boccalatte, Kelly V. Ruggles, Gregory David

**Affiliations:** 1 Department of Biochemistry and Molecular Pharmacology, New York University Grossman School of Medicine, NYU Langone Health, New York, NY 10016 USA; 2 Perlmutter Cancer Center, New York University Grossman School of Medicine, NYU Langone Health, New York, NY 10016 USA; 3 Department of Medicine, New York University Grossman School of Medicine, NYU Langone Health, New York, NY 10016 USA; 4 Department of Pathology, New York University Grossman School of Medicine, NYU Langone Health, New York, NY 10016 USA; 5 Department of Urology, New York University Grossman School of Medicine, NYU Langone Health, New York, NY 10016 USA

**Keywords:** Hematopoiesis, Cell cycle, Differentiation, Stem cells, Chromatin

## Abstract

**Background:**

Blood homeostasis requires the daily production of millions of terminally differentiated effector cells that all originate from hematopoietic stem cells (HSCs). HSCs are rare and exhibit unique self-renewal and multipotent properties, which depend on their ability to maintain quiescence through ill-defined processes. Defective control of cell cycle progression can eventually lead to bone marrow failure or malignancy. In particular, the molecular mechanisms tying cell cycle re-entry to cell fate commitment in HSCs remain elusive. Previous studies have identified chromatin coordination as a key regulator of differentiation in embryonic stem cells.

**Results:**

Here, we utilized genetic inactivation of the chromatin-associated Sin3B protein to manipulate cell cycle control and found dysregulated chromatin accessibility and cell cycle progression in HSCs. Single cell transcriptional profiling of hematopoietic stem and progenitor cells (HSPCs) inactivated for Sin3B reveals aberrant progression through the G_1_ phase of the cell cycle, which correlates with the engagement of specific signaling pathways, including aberrant expression of cell adhesion molecules and the interferon signaling program in LT-HSCs. In addition, we uncover the Sin3B-dependent accessibility of genomic elements controlling HSC differentiation, which points to cell cycle progression possibly dictating the priming of HSCs for differentiation.

**Conclusions:**

Our findings provide new insights into controlled cell cycle progression as a potential regulator of HSC lineage commitment through the modulation of chromatin features.

**Supplementary Information:**

The online version contains supplementary material available at 10.1186/s13072-024-00526-w.

## Background

Hematopoietic stem cells (HSCs) are rare, multipotent, self-renewing adult stem cells responsible for maintaining hematopoiesis [1]. HSCs sit on top of a hierarchically organized system of progenitors that give rise to differentiated blood cells [2]. To maintain stemness, both extrinsic and intrinsic factors enforce a quiescent state in HSCs [3], which is thought to support their long-term fitness by shielding them from environmental damage and cellular injury [4]. Extrinsic factors regulating HSC quiescence stem from their unique localization within the bone marrow. Intrinsic factors dictating HSC quiescence include canonical cell cycle pathways, such as Rb-E2F repression.

The proliferative status of HSCs inversely correlates with their functional potency, as actively cycling cells exhibit diminished reconstitution potential when transplanted into mice [5]. Label retention studies revealed that a subpopulation of dormant, i.e. non-proliferative, HSCs encompasses the entire reconstitution capacity of the stem cell compartment [6]. In response to increased demand, these dormant HSCs enter a primed or activated state that potentiates faster re-entry into the cell cycle to reestablish homeostasis [7, 8]. Activated HSCs may also revert to dormancy, but the molecular mechanisms regulating this reversible switch remain unknown. In addition, how cell cycle exit protects HSCs’ multipotency properties is also unknown.

The contribution of cell cycle position to differentiation has been investigated in other stem cell types, namely embryonic stem cells (ES) [9]. ES cells possess a unique cell cycle structure with a truncated G_1_ phase [10] which is thought to promote self-renewal by limiting exposure to pro-differentiation signals [11]. Accordingly, lengthening the G_1_ phase causes spurious differentiation in ES cells [12] and as ES cells differentiate, their transit time through G_1_ also increases [13]. However, whether location in a discrete cell cycle phase is a cause or consequence of differentiation in ES cells remains unresolved.

The relationship between cell cycle and differentiation in adult stem cells remains ill-defined. HSCs, like other somatic cells, possess a substantial G_1_ phase subject to regulation by the canonical cell cycle machinery. A regulatory function for G_1_ in HSC differentiation has been proposed, based on the observation that CCND1-CDK4 overexpression in human HSCs shortens G_1_ and biases cellular fate towards self-renewal at the expense of differentiation [14]. A role for G_1_ in differentiation is further supported by the demonstration that HSCs can differentiate without undergoing mitosis, suggesting that completing cellular division is dispensable for differentiation in HSCs [15]. Finally, a report has shown that the speed of cell cycle progression in megakaryocytic-erythroid progenitors (MEPs) determines their fate specification, where faster cell cycle progression promotes the erythroid fate over the megakaryocytic fate [16]. Together, these data suggest that cell cycle progression could be a determinant of differentiation. However, how cell cycle machinery affects transcriptional output related to differentiation in HSCs remains unknown.

Genetic experiments to modulate cell cycle usually result in spurious proliferation and exhaustion of HSCs, including p53 deletion [17]. However, some regulators can more subtly alter cell cycle kinetics, especially to regulate the transition between G_0_ and G_1_. To dissect the relationship between cell cycle progression in G_1_ and HSC differentiation, we exploited a mouse strain where the transcriptional repressor Sin3B is genetically inactivated in the hematopoietic system [18]. Sin3B is an evolutionarily conserved non-catalytic component of the Sin3 transcriptional repressor complex [19–21]. Through its interaction with sequence-specific transcription factors, Sin3B tethers histone repressors, including histone deacetylases (HDACs) and histone demethylases (HDMs) to discrete genomic loci.

The transcriptional state of any gene is not only dependent on the transcriptional machinery and associated co-factors, but also on a set of discrete post-translational modifications at nucleosomes, which modulates DNA accessibility [22]. Active transcription depends on a balanced action of chromatin remodelers and histone modifiers, the latter including histone acetyltransferases and histone deacetylases [23]. Specifically, histone deacetylases (HDACs) are a family of enzymes that remove acetyl moieties from target lysines [24]. At promoters, the HDAC-mediated deacetylation of lysine residues within N-terminal tails of histones H3 and H4 generally correlates with active transcriptional repression, while acetylation correlated with transcriptional activation. Many of these enzymes do not have DNA-binding activity and are therefore recruited by large multi-subunit complexes to specific genomic loci [25].

We have shown that Sin3B is a key contributor to cell cycle exit in numerous biological contexts [26–28], with a portion of Sin3B’s pro-quiescence function being mediated in part through its interaction with the Dimerization Partner (DP), Retinoblastoma (RB)-like, E2F, and MuvB (DREAM) complex, a critical regulator of quiescence and G_1_ entry [29]. We had previously observed that in a T98G glioma cell line, about one-third of genes derepressed upon Sin3B genetic ablation were determined to be DREAM targets by bulk RNA-Seq[29]. In addition, we had previously observed increased acetylation at the promoter of cell cycle target genes in quiescent conditions upon Sin3B inactivation, suggesting that DREAM and Sin3B cooperate, at least in part, to control cell cycle exit [29]. Genetic inactivation of Sin3B in the hematopoietic system via the Vav1-iCre transgene impairs HSCs' quiescence and abolishes their ability to reconstitute the hematopoietic system in a competitive transplantation setting but does not affect their self-renewal and survival [18]. Thus, Sin3B inactivation uncouples self-renewal and differentiation in HSCs and offers a unique opportunity to interrogate the relationship between cell cycle progression and differentiation.

Our results identify Sin3B as both an essential gatekeeper of early cell cycle progression in HSCs, and a molecular switch for HSC lineage commitment through the modulation of chromatin accessibility at cis-regulatory elements driving hematopoietic differentiation. We demonstrate that loss of Sin3B results in spurious progression through the early phases of the cell cycle, yet we only detected nominal changes in the expression of non-cell-cycle related genes by single cell RNA-Seq. However, we did identify changes to accessibility of differentiation related enhancers in Sin3B-null LT-HSCs by ATAC-Seq. These results point to controlled progression through the G_1_ phase of the cell cycle as a potential regulator of HSC lineage commitment through regulation of chromatin features.

## Results

### Genetic inactivation of Sin3B results in LT-HSC expansion and aberrant transcriptional signatures

We have previously demonstrated that Sin3B^−/−^ whole bone marrow cells (WBM) cannot reconstitute the hematopoietic system of lethally irradiated mice in a competitive transplantation at 20 weeks [18]. We have found that recipient mice showed minimal contribution of Sin3B^−/−^ cells to all hematopoietic lineages assayed in peripheral blood by flow cytometry. At 20 weeks, when the hematopoietic system has returned to homeostasis, we analyzed the bone marrow to determine the contribution of donor cells to the stem cell compartment. To analyze cellularity in the bone marrow, we employed a labeling strategy utilizing expression of Lineage markers, Sca-1, cKit, Flk2, CD48, and CD150 to distinguish between HSCs, with LT-HSCs being the most primitive stem cells, and Multipotent Progenitors (MPPs), that are more lineage restricted (Additional file 2: Fig. S1). The analysis revealed that Sin3B^−/−^ Long Term (LT)-HSCs are present in the bone marrow in comparable proportions to their wild-type (Sin3B^+/+^)counterparts, despite minimal contributions to the peripheral blood. We also have demonstrated that these cells were not apoptotic and were able to correctly home to the bone marrow of recipient mice [18].

**Fig. 1:**
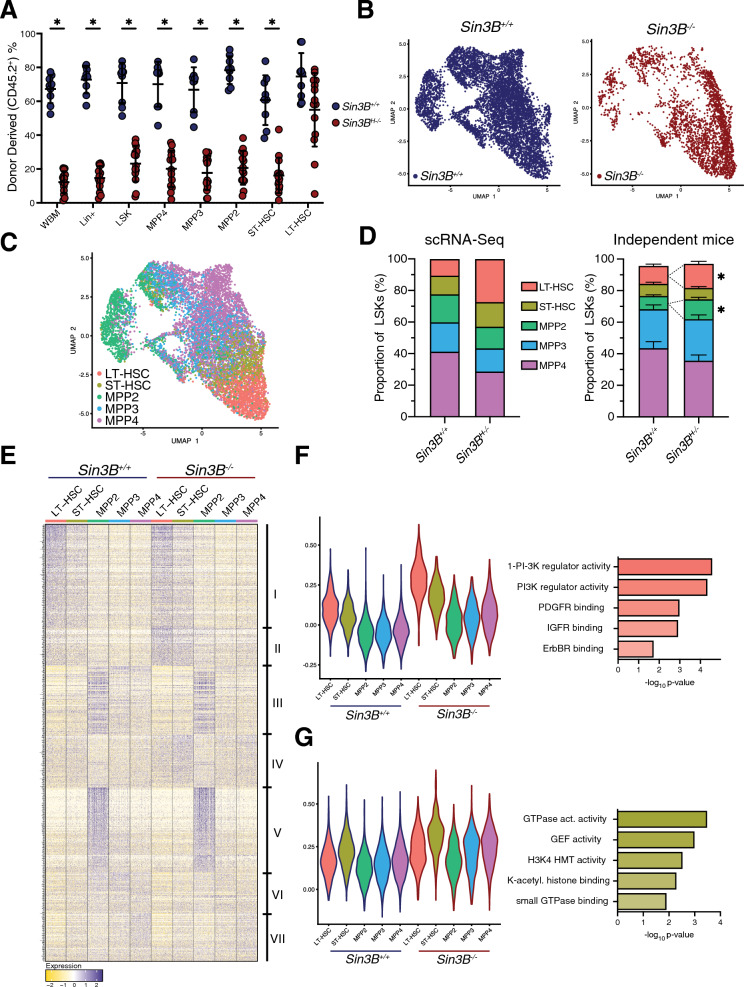
Sin3B regulates discrete transcriptional programs in hematopoietic subsets at homeostasis. **A** Analysis of whole bone marrow from recipient mice 8 weeks after competitive transplantation. Quantification of donor derived (CD45.2) cells in the hematopoietic compartments indicated via flow cytometry. Asterisks indicate statistical significance. (2way ANOVA, Šidák’s multiple comparison’s test; *p < 0.0001; Data are represented as mean ± SEM) Sin3B^F/F^ recipients *n* = 9; Sin3B^−/−^ recipients *n* = 14. **B** Two-dimensional Uniform Manifold Approximation and Projection (2D UMAP) with LSK cells colored by genotype as determined by hashtag oligo analysis. Left: Sin3B^+/+^ cells. Right: Sin3B^−/−^ cells. LSKs were sorted from 4 mice, 2 for each genotype. **C** 2D UMAP demonstrating supervised clustering of hematopoietic subsets recovered from scRNA-Seq. **D** Left panel: quantification of various LSK subsets in scRNA-Seq. Right panel: Quantification of same subset in the bone marrow of independent mice by flow cytometry. Asterisks indicate statistical significance (Student’s t-test with multiple comparison correction; *p < 0.05; data shown as mean ± SEM) (*n* = 9). **E** Differentially expressed genes between all cell types was calculated via Seurat’s FindAllMarkers function utilizing the MAST statistical framework. Clusters of genes visually identified are denoted on the right. Expression is scaled and centered. **F** Left panel: Violin Plot showing average expression of Cluster II genes for indicated hematopoietic subtypes. Right panel: Gene Ontology Analysis of Cluster II genes. **G** Left panel: Violin Plot showing average expression of Cluster IV genes for indicated hematopoietic subtypes. Right panel: Gene Ontology Analysis of Cluster IV genes

To determine if Sin3B^−/−^ LT-HSCs are unable to differentiate or exhibit a delayed response we analyzed recipient mice 8 week post-transplantation, when LT-HSCs are actively engaged in differentiation. Sin3B^−/−^ WBM contributed minimally to total marrow cellularity (16%) compared to Sin3B^+/+^ (67%) (Fig. 1A). This pattern held for all hematopoietic subtypes (LSKs, MPP2s, MPP3s, MPP4s, ST-HSCs) with limited self-renewal [24]. By contrast, both Sin3B^+/+^ and Sin3B^−/−^ cells contributed to the LT-HSC compartment (Fig. 1A). These data indicate that Sin3B^−/−^ LT-HSCs can survive, proliferate, and self-renew in a transplantation setting, but are impaired in their ability to differentiate.

To delineate the differentiation defect in Sin3B^−/−^ LT-HSCs, we performed single-cell RNA-Seq on Lineage^−^Sca-1^+^cKit^+^ (LSK) cells from Sin3B^+/+^ and Sin3B^H−/−^ mice (*Sin3B*^*F/F*^*; Vav1-iCre*^+^) and obtained data for 9586 cells. We assigned genotypes to each cell (Fig. 1B) [30] and subjected the dataset to supervised clustering using previously published transcriptional signatures [31, 32] (Fig. 1C). Our quality control analysis included removing cells that expressed any lineage-related genes, as we wanted to remove cells that our sorting strategy was not able to exclude, which only accounted for a couple dozen cells in the entire dataset. We recovered all five hematopoietic subsets (LT-HSC, ST-HSC, MPP2, MPP3, MPP4) in both genotypes, consistent with the lack of an overt phenotype in Sin3B^H−/−^ mice at homeostasis [18]. LT-HSCs contain the greatest differentiation potential and extensive self-renewal capabilities, as well as exhibiting the lowest levels of cell cycle activity [4]. LT-HSCs give rise to ST-HSCs, which share common properties with LT-HSCs, but are not able to self-renew to the same extent. These ST-HSCs then give rise to more lineage restricted MPPs, with MPP2’s biased towards a megakaryocyte-erythroid lineage, MPP3’s exhibiting a myeloid bias, and MPP4’s being biased towards the lymphoid lineage [25]. We observed that LT-HSCs cluster near ST-HSCs, which separate LT-HSCs from the MPP subsets. Given the heterogeneity in these populations, and the transcriptional similarity between different HSPC subsets, we noted close clustering of these cells, similar to other published works [33–35]. We noted an increase in the proportion of LT-HSCs of Sin3B^H−/−^ mice (27% vs 10%) (Fig. 1D, left panel), and we confirmed this result in an independent cohort of mice (Fig. 1D, right panel), corroborating the expansion of the LT-HSC compartment in Sin3B^H−/−^ mice at homeostasis.

Next, we sought to identify the transcriptional programs affected by Sin3B loss in each hematopoietic subtype. We grouped 842 differentially expressed genes into 7 clusters based on expression patterns across genotypes, cell identity, or both (Fig. 1E). We highlighted two clusters of interest: Cluster II contained 74 genes that display the highest expression in LT-HSCs and are uniformly upregulated upon Sin3B loss in LSKs (Fig. 1F, left panel). Gene Ontology (GO) analysis points to PI3K regulation. Previous reports had highlighted PI3K signaling as a mediator of HSC activation upon stress [36]. Cluster IV comprised 102 genes enriched in GTPase activity (Fig. 1G) [37]. These genes are upregulated in Sin3B^−/−^ cells across different subtypes, with highest expression in ST-HSCs, highlighting a role in the LT- to ST-HSC transition. In particular, the Rho GTPase Cdc42 has recently been implicated in regulating symmetric vs asymmetric divisions in LT-HSCs [38]. These clusters indicate that we can capture stereotyped transcriptional changes as LT-HSCs differentiate into ST-HSCs and subsequent MPP subsets.

### A Sin3B-dependent transcriptional program is engaged upon the transition from LT- to ST-HSCs at homeostasis

**Fig. 2:**
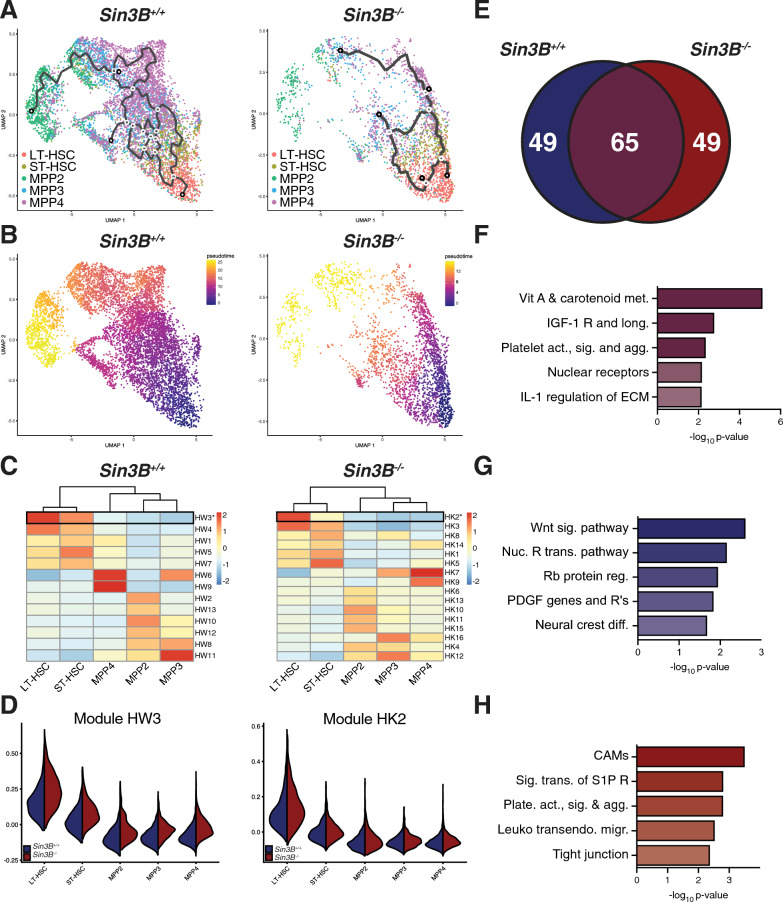
Pseudotime analysis of LSKs at homeostasis demonstrates a defective transition between LT- and ST-HSCs in the absence of Sin3B. **A** Two-dimensional UMAP projection of LSKs showing trajectories calculated from Monocle3. Left panel: Sin3B^+/+^, Right panel: Sin3B^−/−^. **B** Using LT-HSCs as a starting node, pseudotime was calculated for each genotype to determine transcriptional programs as a function of differentiation. **C** Differentially expressed genes as a function of pseudotime were calculated and grouped into modules based on expression pattern. Shown is the average expression of each module in the indicated cell type. **D** Expression of highlighted modules in Sin3B^+/+^ and Sin3B^−/−^ subsets. **E** Venn diagram showing overlap of genes obtained from module HW3 and HK2. Purple color denotes overlap, blue denotes wild-type, and red denotes knockout. **F** Gene Ontology Molecular Function was carried out for the indicated gene list and shown are GO terms recovered plotted by p-value for 65 genes shared bewteen Sin3B^+/+^ and Sin3B^-/-^ LT-HSCs. **G** GO Molecular Function was carried out for 49 genes unique to Sin3B^+/+^ LT-HSCs. **H** GO Molecular Function was carried out for 49 genes unique to Sin3B^−/−^ LT-HSCs

We next analyzed how the differentiation program from LT-HSC to MPPs is altered upon Sin3B loss. We subjected the dataset to trajectory analysis using the Monocle3 package [39]. We separated the cells by genotype to capture the differentiation trajectory in Sin3B^+/+^ cells (Fig. 2A, left panel). This procedure captures transcriptional paths as cells transition between cell types. The interconnectedness of the cells resembles CLOUD-HSPCs [40]; a recent report that postulated stem and progenitor cells exist along a continuum of low-priming and gradually gain lineage commitment. The same analysis performed on Sin3B^−/−^ LSKs (Fig. 2A, right panel) revealed a decrease in the number of trajectories, denoting fewer differentiation opportunities. While we did have less Sin3B^−/−^ cells, we did have an enrichment of LT-HSCs, with the other cell type proportions being comparable, and yet the structure of the data, especially in the transition from LT- to ST-HSCs was much different in the Sin3B^−/−^compared to the Sin3B^+/+^. We separated the trajectory analysis, in order to capture the normal movement of cells between different states as they differentiated and to avoid calculating artificial trajectories that represented hybrid states that were averages of Sin3B^+/+^ and Sin3B^−/−^ data when combined. We then postulated that we could identify the position of the block in differentiation using Sin3B^−/−^ data.

Next, we delineated a pseudotime utilizing a branch point in the LT-HSC cluster as an anchor to capture a path from LT-HSCs, through ST-HSCs, then to MPPs (Fig. 2B). We calculated gene expression changes as a function of pseudotime and grouped genes based on expression patterns, we reasoned that this could be used as a proxy for gene changes as a function of differentiation. The differentiation block in Sin3B^−/−^ cells occurs at the level of LT-HSCs, so we documented the changes in expression from LT-HSCs to ST-HSCs in our Sin3B^+/+^ dataset, and then investigated perturbations upon Sin3B loss. We identified a module of genes for each genotype that displayed the highest average gene expression in LT-HSCs and decreased as cells progressed to MPPs (Fig. 2C, D). We compared these modules directly to identify a unique transcriptional signature that could explain the phenotype in LT-HSCs elicited upon Sin3B loss (Fig. 2E).

We identified 114 genes in each module, and Gene Ontology (GO) Analysis of the 65 genes commonly downregulated in LT-HSCs revealed an enrichment for Vitamin A metabolism (Fig. 2F), which was noteworthy given the recently described role for retinoic acid in dormancy of LT-HSCs [41]. These data suggest both Sin3B^+/+^ and Sin3B^−/−^ LT-HSCs properly disengage from the dormancy program. Next, we focused on the transition between Sin3B^+/+^ LT- and ST- HSCs, which would represent the normal differentiation trajectory. The 49 genes uniquely expressed in Sin3B^+/+^ LT-HSCs showed an enrichment for the Wnt signaling pathway and in Rb protein regulation (Fig. 2G). Wnt signaling has been known to contribute to multiple aspects of developmental hematopoiesis [42]. The over-representation of the Rb pathway in Sin3B^+/+^ cells is consistent with the previously published role for Sin3B in modulating Rb-E2F transcriptional repression [19, 27, 29]. Analyzing the 49 genes that were uniquely expressed in Sin3B^−/−^ LT-HSCs, we identified pathways enriched for various cell–cell interaction molecules, with Cell Adhesion Molecules registering as the most significantly enriched term (Fig. 2H). Given the importance of adhesion and migration in HSCs within the niche and their impact on differentiation [43], it is tempting to speculate that the differentiation block elicited upon Sin3B inactivation stems in part from a defective adhesion and/or migration of LT-HSCs within the niche. Further work will need to be done to demonstrate a definitive link between niche migration and differentiation in cells with altered cell cycle kinetics.

### Sin3B loss results in aberrant LT-HSC stress responses upon challenge with 5-FU

The scRNA-Seq analysis described above confirmed the subtle transcriptional deregulation of the LT-HSC compartment in Sin3B^H−/−^ mice. However, the Sin3B^−/−^ HSC phenotype only manifests during a stress response, when HSCs are actively engaging in differentiation. To determine the Sin3B-dependent transcriptional changes in response to stress, we performed scRNA-Seq on LSKs isolated from Sin3B^F/F^ and Sin3B^H−/−^ mice 9 days after 5-Fluororuracil (5-FU) administration. 5-FU induces myelosuppression and subsequent activation of LT-HSCs to compensate for the elimination of rapidly proliferating progenitors that maintain homeostasis. The number of HSCs peaks nine days after 5-FU administration before returning to homeostasis [44], at which point HSCs are engaged in differentiation. We reasoned that by transcriptionally profiling cells at this point, we could capture the differentiation block during a stress response and better understand why Sin3B^−/−^ cells do not contribute to peripheral blood populations or bone marrow, despite their ability to self-renew.

**Fig. 3:**
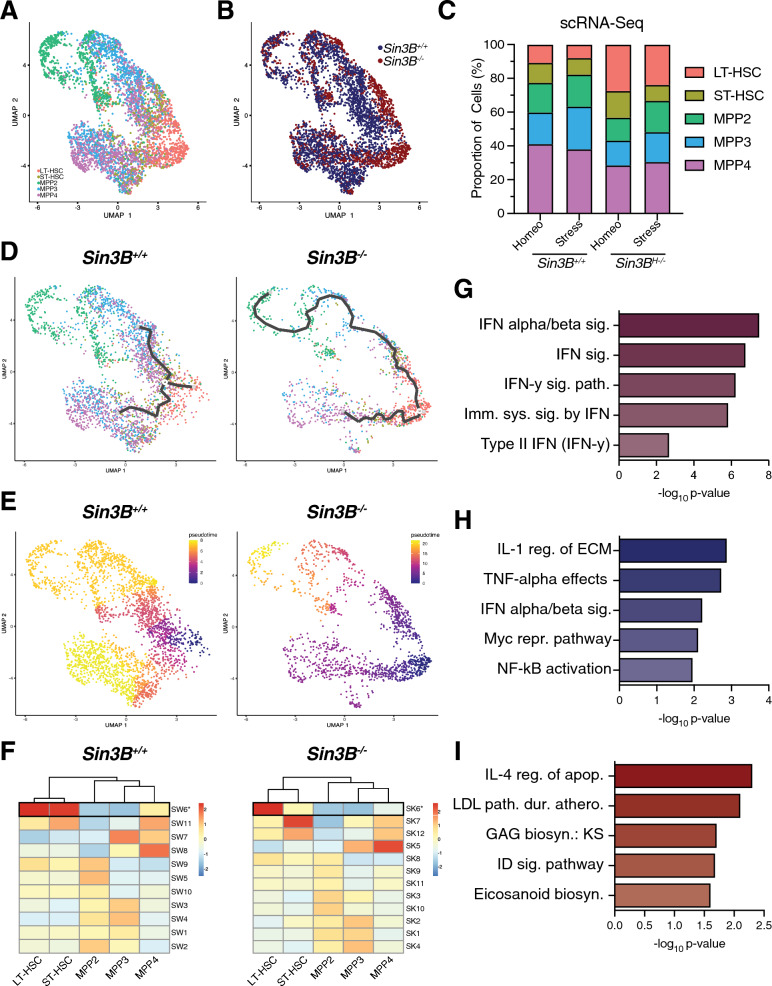
Sin3B^−/−^ LSKs display defective stress hematopoiesis. **A** LSKs were sorted from Sin3B^F/F^ and Sin3B^H−/−^ mice 9 days after 5-FU administration (100 mg/kg, i.p.) and subjected to scRNA-Seq analysis utilizing the same strategy as in Fig. 1. *n* = 2 mice per genotype. **B** Sin3B^+/+^ and Sin3B^−/−^ cells were able to be separated in our data based on hashtag oligonucleotide sequencing. **C** Quantification of different hematopoietic subsets at indicated conditions. **D** Monocle3 differentiation trajectory analysis of Sin3B^+/+^ and Sin3B^−/−^ LSKs during a stress response. **E** Pseudotime analysis utilizing LT-HSCs as starting node for either Sin3B^+/+^ and Sin3B^−/−^ LSKs. **F** Differential expression analysis was used to calculate modules of genes grouped together based on expression as a function of pseudotime. **G** Gene Ontology analysis for 100 genes in common. **H** Gene Ontology analysis for 136 genes unique to Sin3B^+/+^ LT-HSCs. **i** Gene Ontology analysis for 126 genes unique to Sin3B^−/−^ LT-HSCs

We applied the same computational approach to this dataset as for LSKs at homeostasis (Fig. 2) and recovered 4032 cells and assigned a genotype and hematopoietic subset identity to each cell (Fig. 3A, B). When compared to homeostasis, we observed a contraction in the stem cell compartment of Sin3B^+/+^ mice exposed to stress, consistent with previous reports showing that in regenerative conditions, MPP2s and MPP3s expand to replenish myeloid output, with lymphoid regeneration taking place later [31]. We noted a greater expansion of the stem cell compartment in Sin3B^H−/−^ mice compared to their Sin3B^+/+^ counterparts (Fig. 3C).

We again applied a trajectory analysis to determine the transcriptional changes in LT-HSCs as they transition to ST-HSCs and subsequent MPPs. Monocle3’s trajectory analysis revealed a more direct differentiation process in stress conditions than at homeostasis, specifically with LT-HSCs transitioning directly to MPP’s in both Sin3B^+/+^ and Sin3B^−/−^ cells, with fewer ST-HSCs in the space between LT-HSCs and MPP subtypes (Fig. 3D). Next, we determined a pseudotime for each genotype (Fig. 3E), again reasoning that this path linking LT-HSCs to MPPs could serve as a proxy for the transcriptional changes during differentiation. We identified two gene sets with the highest expression in LT-HSCs that decreased as cells transitioned to MPPs, which included 136 genes in the Sin3B^+/+^ data (Modules SW6) and 126 genes in the Sin3B^−/−^ data (Modules SK6) (Fig. 3F). The overlap between these modules revealed that 100 genes were downregulated as LT-HSCs transitioned into MPPs in both Sin3B^+/+^ and Sin3B^H−/−^ mice. GO analysis indicated an enrichment for interferon signaling (Fig. 3G), consistent with previous studies of stress hematopoiesis [45]. This suggests that both Sin3B^+/+^ and Sin3B^−/−^ LT-HSCs can sense local inflammatory conditions. The 36 genes unique to wild-type LT-HSCs showed an enrichment for the NF-κB pathway (Fig. 3H), which is known to be engaged downstream in response to hematopoietic insults [46]. This group of genes also displayed an enrichment for the IFN α/β signaling pathway, suggesting that while IFN signaling is engaged upon stress in Sin3B^−/−^ LT-HSCs, the intensity or the integrity of this pathway is altered in these conditions, as evidenced by the lack of TNF signaling in Sin3B^−/−^ LT-HSCs, despite expressing genes in the IFN pathway. Finally, the 26 genes unique to Sin3B^−/−^ LT-HSCs exhibited no consistent function as a group (Fig. 3I). Together, these data suggest that Sin3B^−/−^ LT-HSCs can sense stress due to 5-FU treatment but are unable to engage the appropriate differentiation programs. Of note, Sin3B^−/−^ LT-HSCs do not downregulate genes within the NF-κB pathway upon stress, thus providing a potential mechanism for their block in differentiation in this context.

### Sin3B restricts progression along the G_1_ phase of the cell cycle in LT-HSCs

To understand the transcriptomic misregulation of differentiation upon loss of Sin3B, we first assessed the expression of master transcription factors responsible for differentiation of various lineages [47]. We found no change in expression of these transcription factors upon loss of Sin3B (Additional file 3: Fig. S2). With the seemingly normal expression of components of the differentiation machinery in HSCs, we hypothesized that aberrant cell cycle progression may contribute to the inability of Sin3B^−/−^ LT-HSCs to differentiate, considering the previously characterized Sin3B-driven repression of cell cycle genes.

**Fig. 4:**
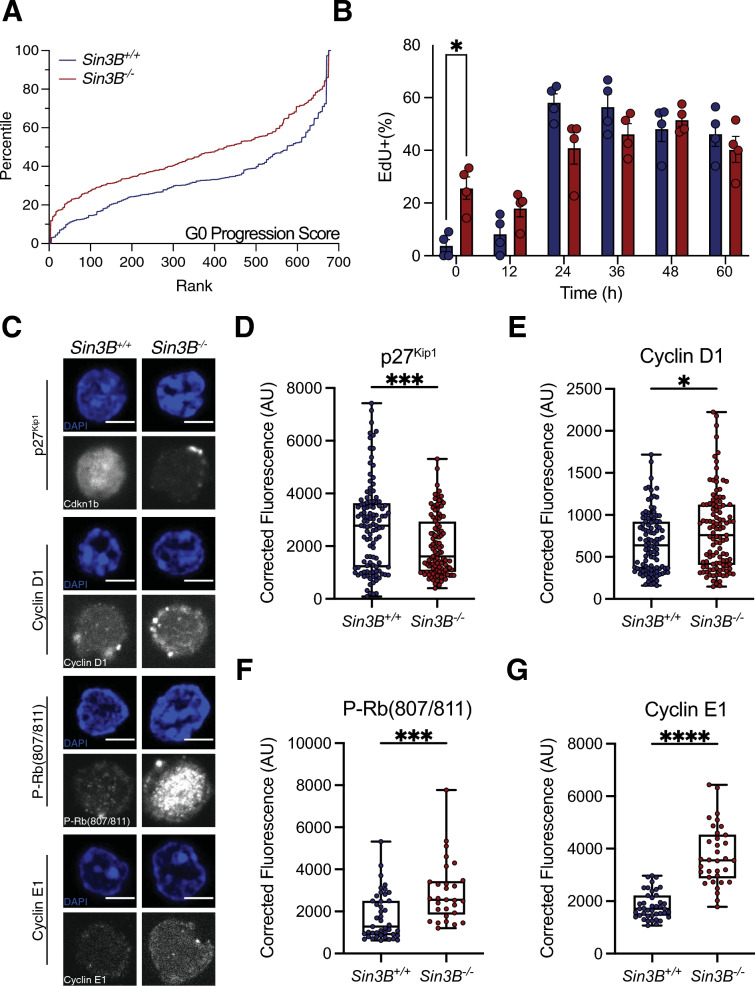
Ablation of Sin3B results in spurious cell cycle progression in LT-HSCs. **A** G_0_ scores for LT-HSCs were calculated and cells were ranked and data transformed into percentile to normalize for cell number. **B** EdU labeling of cycling LT-HSCs from indicated genotypes at various timepoints via immunofluorescence. Asterisks indicate statistical significance (2-way ANOVA, Šidák’s multiple comparison’s test; *p < 0.05; data shown as mean ± SEM. *n* = 4 per genotype. **C** LT-HSCs were isolated from Sin3B^F/F^ or Sin3B^H−/−^ animals via FACS and processed for immunofluorescence and quantification of indicated cell cycle proteins. Scale bars represent 5 µm. Signal was quantified within the nucleus of individual cells utilizing DAPI as a mask. Representative immunofluorescence from sorted LT-HSCs of indicated proteins. Images were acquired on a Zeiss LSM 700 Laser Scanning Confocal using a 63× plan apochromat 1.4 oil objective mounted with oil at room temperature. Alexa Flour 488 and Alexa Fluor 594 were the fluorochomes conjugated to primary antibodies against indicated target protein. Zen software was utilized on the microscope to acquire images, with analysis done in FIJI to measure fluorescence intensity. All fluorescence was corrected for using background readings and data was exported to Prism9 for statistical analysis. **D** Quantification of Cdkn1b(p27^Kip1^) in LT-HSCs. **E** Quantification of Cyclin D1 in LT-HSCs. **F** Quantification of Phospho-Rb(S807/811) in LT-HSCs. **G** Quantification of Cyclin E1 in LT-HSCs. Outliers were identified with the ROUT method (*Q* = 1%). Asterisks indicate statistical significance (2-tailed unpaired *t*-test; *p < 0.05; ***p < 0.0001; ****p < 0.00001). *n* = 4 mice per genotype

To test this hypothesis, we first compared the scRNA-Seq data for Sin3B^−/−^ LT-HSCs to a recently published transcriptomic signature for quiescence [48]. Cells were ordered by expression levels and ranked by cell cycle progression. Sin3B loss resulted in a shift in the progression from quiescence towards G_1_ (Fig. 4A), indicative of an impaired cell cycle control in Sin3B^−/−^ LT-HSCs. Next, we tested whether this spurious progression into the cell cycle bore functional consequences, i.e. commitment to cell cycle and transition to S phase. Culture conditions for HSCs contain supraphysiological levels of cytokines and growth factors that enforce HSC cycling. Sin3B^−/−^ LT-HSCs displayed higher levels of EdU incorporation than their wild-type counterparts after 1 h or 12 h in culture, suggesting that Sin3B^−/−^ LT-HSCs are poised to reenter S-phase faster than wild-type cells when cultured in pro-proliferative conditions (Fig. 4B). Furthermore, Sin3B loss resulted in lower levels of p27^Kip1^ expression in LT-HSCs, suggesting that they are less quiescent than their wild-type counterparts (Fig. 4C, D). Next, we detected elevated levels of Cyclin D1 in Sin3B^−/−^ LT-HSCs (Fig. 4C, E), along with increased Rb (S807/811) phosphorylation in Sin3B^−/−^ LT-HSCs (Fig. 4C, F). Assaying for Cyclin E protein levels, a key determinant of the G_1_/S phase transition, we observed an increase in Sin3B^−/−^ LT-HSCs (Fig. 4C, G). In sum, these data indicate that Sin3B restricts LT-HSCs’ progression through G_1_ phase of the cell cycle and Sin3B^−/−^ LT-HSCs are poised to cross the G_1_/S checkpoint faster than their wild-type counterparts when stimulated.

### Loss of Sin3B results in an aberrant chromatin environment that is restrictive to differentiation

Our central hypothesis is that aberrant cell cycle progression directly hinders LT-HSCs to differentiate. Sin3B is an integral component of an HDAC-containing complex, and we therefore hypothesize that Sin3B loss alters the chromatin landscape in a cell-cycle dependent manner that enables the transcriptional machinery to engage expression of the differentiation program in response to stress. To determine the impact of Sin3B on the chromatin accessibility landscape of HSCs, we performed Assay for Transposase-Accessible Chromatin (ATAC)-Seq on purified LT-HSCs from Sin3B^+/+^ and Sin3B^−/−^ LT-HSCs at homeostasis [49].

**Fig. 5:**
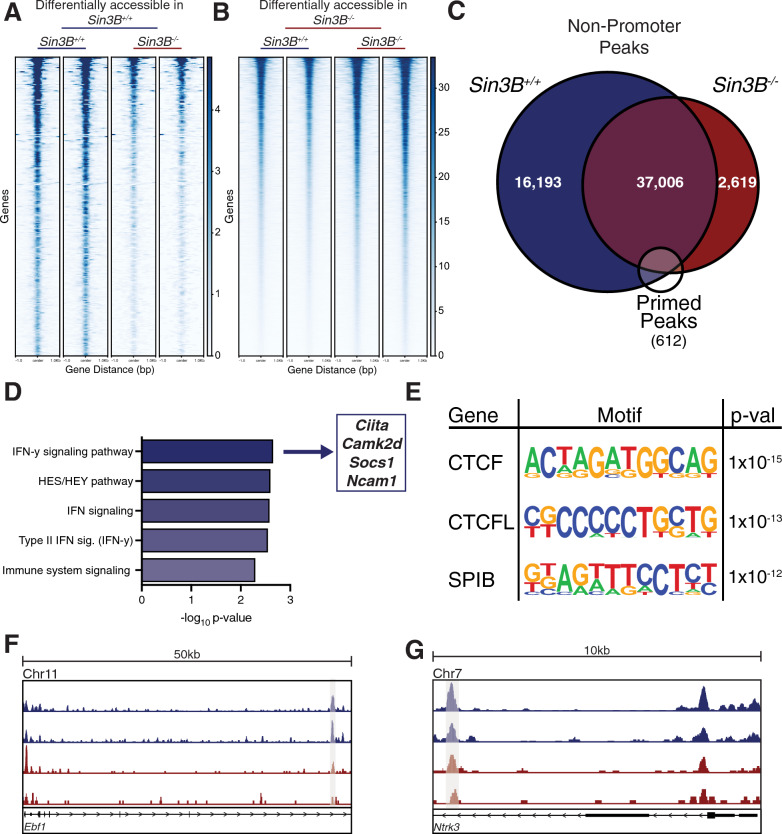
Sin3B promotes a permissive environment for differentiation. ATAC-Seq was performed on isolated LT-HSCs purified via FACS. Differentially accessible regions were calculated using the Diffbind package in R. **A** Heatmap of differentially accessible peaks in Sin3B^F/F^ LT-HSCs. Differential accessibility refers to statistically significant peaks between genotypes. Shown are 287 peaks identified as more open in Sin3B^F/F^ LT-HSCs. Each column represents peaks from one replicate (2 mice) of the indicated genotype. **B** Heatmap of differentially accessible peaks in Sin3B^−/−^ LT-HSCs. Shown are 5531 peaks identified as more open in Sin3B^−/−^ LT-HSCs. **C** Venn diagram showing overlap of non-promoter peaks from dataset, overlapped with a list of published primed peaks from Martin, et al. **D** Primed peaks unique to wild-type LT-HSCs were analyzed with the GREAT tool and the list of genes that are putatively regulated by these regions were analyzed using Enrichr. Shown are the pathways identified by BioPlanet as significantly enriched. **E** Homer motif analysis for primed peaks unique to Sin3B^+/+^ LT-HSCs. Shown are the top 3 motifs and p-values. **F** Representative tracks for the gene Ebf1, a gene regulated by one of the primed peaks identified in (**D**) the cis-regulatory element is highlighted. **G** Representative tracks for the gene Ntrk3, a gene regulated by one of the primed peaks identified in (**D**) the cis-regulatory element is highlighted. Each track represents data from one experimental point

Our analysis identified 64,744 peaks corresponding to accessible chromatin regions in Sin3B^+/+^ LT-HSCs, and 50,655 peaks in Sin3B^−/−^ LT-HSCs. Interestingly, Sin3B^−/−^ LT-HSCs displayed overall higher levels of accessibility relative to their Sin3B^+/+^ counterparts, despite having fewer of these sites (Additional file 4: Fig. S3). This observation is consistent with Sin3B’s known function in coordinating the recruitment of histone repressors to discrete genomic loci [19, 29]. Using Diffbind in R to identify differentially accessible peaks utilizing a DESeq2 workflow with an FDR < 0.05 between the Sin3B^+/+^ and Sin3B^−/−^ LT-HSCs [50], we uncovered 287 peaks that are more accessible in Sin3B^+/+^ LT-HSCs, compared to 5531 accessible peaks that are more accessible in Sin3B^−/−^ LT-HSCs (Fig. 5A, B). In agreement with the scRNA-Seq data, we found that over 40 genes we previously determined to be upregulated in Sin3B^−/−^ LT-HSCs displayed a concomitant increase in chromatin accessibility by ATAC-Seq in these cells (Additional file 6: Fig. S5).

Next, we annotated peaks using HOMER to determine the genomic features of the loci whose accessibility is modulated by Sin3B [51]. We found no discernable differences in the respective proportion of any given genomic feature accessible in Sin3B^+/+^ and Sin3B^−/−^ LT-HSCs, such as the 5’ UTR and exons (Additional file 5: Fig. S4A). Strikingly, the proportion of genomic features more accessible in Sin3B^−/−^ LT-HSCs exhibited a significant enrichment in promoter regions, at the expense of intergenic regions (Additional file 5: Fig. S4B, C). As these regions are enriched for regulatory sequences, we reasoned that the differentiation defect we observe in Sin3B^−/−^ LT-HSCs may result from defective enhancer engagement. To test this, we compared a list of “primed” peaks [52], which correspond to enhancers that are present in an active chromatin state in LT-HSCs, and in specific lineages of peripheral blood cells, with the putative regulatory regions identified as more accessible in Sin3B^+/+^ or in Sin3B^−/−^ LT-HSCs (Fig. 5C). We identified 493 primed peaks in Sin3B^+/+^ LT-HSCs, and 445 primed peaks in Sin3B^−/−^ LT-HSCs, and found 64 peaks that were unique to Sin3B^+/+^ LT-HSCs (Fig. 5C, F, G). We used the GREAT tool to interrogate which genes these 64 enhancers regulate and uncovered over 100 putatively regulated genes. GO analysis for this group of genes revealed significant enrichment for interferon gamma signaling (Fig. 5D), consistent with our previous identification of interferon signaling as a transcriptional signature altered in stress conditions in our scRNA-Seq (Fig. 3H, I) [53]. In addition, a report by Baldridge and colleagues demonstrated that interferon gamma was important in mediating LT-HSC activation and proliferation [54]. Together, these results suggest a defect in the chromatin accessibility of loci related to interferon signaling in Sin3B^−/−^ LT-HSCs, which correlates with their commitment to downstream lineages and subsequent differentiation.

Next, we identified the putative sequence-specific transcription factors bound to the 64 primed peaks that were unique to Sin3B^+/+^ LT-HSCs. The top two enriched motifs corresponded to the binding sites for CTCF and the related CTCFL/BORIS (Fig. 5e). These proteins have been implicated in maintaining quiescence in hematopoietic stem cells in mice, and their inactivation results in defective survival and differentiation in HSCs [55]. In addition, Qi and colleagues demonstrated that CTCF mediates cis-regulatory element binding with their promoters as HSCs differentiate into a specific lineage, with no impact on TAD formation or boundaries [56]. Our results indicate that the accessibility to CTCF binding sites that control HSC differentiation is impaired in the absence of Sin3B. As previous studies have demonstrated that dynamic CTCF binding is required for lineage specific differentiation in human HSCs, to mediate the transition into “activation” in human HSPCs, it is tempting to speculate that the differentiation of Sin3B^−/−^ HSCs is functionally linked to the compromised accessibility of these CTCF binding sites.

## Discussion

HSCs must balance differentiation capabilities with self-renewal and proliferation potential to maintain stemness features. Proliferation and self-renewal require re-entry into the cell cycle, making early cell cycle progression an intrinsic mechanism of HSC function. We demonstrate here that the Sin3B protein potentiates differentiation in HSCs, correlating with its ability to restrict progression within the G_1_ phase of the cell cycle. This is supported by data showing a spurious progression into G_1_ in HSCs genetically inactivated for Sin3B. Our evidence suggests that Sin3B fine tunes mechanisms of HSC differentiation, and its loss results in an inability of the cell to regulate differentiation.

We have previously shown by bulk RNA-Seq in serum starved T98G cells, that Sin3B exerts the repression activity for only a subset of DREAM targets [29]. Both Sin3B and DREAM have non-overlapping targets, some of which are involved in modulating cell cycle progression. Interestingly, Barret et al. recently reported similar findings in serum starved or quiescent cells, in that they observe a de-repression of DREAM targets in Sin3B-null cells [57]. This Sin3B-dependent repression may be limited to specific biological situations, including serum starvation and other quiescence settings. We propose here that HSC quiescence represents one such biological setting where Sin3B contributes to the repression of cell cycle genes. In addition, we posit that genes involved in differentiation are regulated in a cell cycle specific manner, namely, that progression through the cell cycle causes changes to chromatin structure that alter accessibility.

It has been previously demonstrated that embryonic stem cells exhibit varied levels of response to differentiation stimuli depending on their cell cycle phase [11]. Data from ES cells have demonstrated that after mitosis, specific regulatory sequences driving self-renewal and stemness contact their target genes earlier in G_1_, unlike regulatory sequences controlling differentiation [58]. These observations suggest a prioritization of chromatin unfolding whereby the spatial organization of genes related to cellular identity is quickly re-established before other transcriptional programs can be engaged. Once cells commit to proliferation and initiate S phase, the chromatin landscape is restructured to prepare for genome replication. Here, we postulate the existence of a “commitment window” that begins when chromatin unfolds after mitosis and ends when chromatin is reconfigured for DNA replication.

Classically, the dogma regarding chromatin accessibility in stem cells was that more primitive cell types exhibit open chromatin related to the various lineages they could differentiate into, and as cell fate was specified, chromatin accessibility is gradually lost as stem cell reduce their pluripotency [59]. This model has been recently challenged in various cell types, particularly in HSCs where some regulatory regions of the genome close as HSCs differentiate, and many enhancers are functionally established de novo in more downstream progenitors [60]. In addition, it is thought that HSCs exist along a continuum, with stochastic events causing individual stem cells to become primed to a particular lineage [61]. We posit that the ability to become primed for a particular lineage is in part determined by the position within the cell cycle, with HSCs being able to remodel their chromatin in response to differentiation signals within G_0_ or early G_1_. Our ATAC-Seq data highlights the loss of some primed peaks upon Sin3B inactivation, with Sin3B^−/−^ LT-HSCs being located further along in the G_1_ phase compared to their wild-type counterparts. In addition, we did not detect changes in the expression of various transcription factors that would bind to these regulatory regions, pointing to chromatin accessibility as a likely limiting factor for lineage-related gene expression. We speculate that only the enhancers related to differentiation are affected by the cell cycle deregulation, as we do not observe changes in the expression of self-renewal genes in Sin3B^−/−^ LT-HSCs, which remain able to self-renew and proliferate in vivo.

This hypothesis is further supported by data showing that overexpression of CCND1-CDK4 in human HSCs confers a competitive advantage in vivo, as they transition from G_0_ to G_1_, and are refractory to differentiation signals in vitro [14]. In addition, as a result of spurious cell cycle progression, Sin3B^−/−^ LT-HSCs display aberrant transcriptional programs related to differentiation, such as their inability to downregulate the Wnt pathway as they transition to ST-HSCs, and their improper expression of cell adhesion molecules. Of note, Sin3B^−/−^ LT-HSCs appear to improperly express and regulate some genes in the interferon pathway (Fig. 3G), consistent with an impaired chromatin accessibility profile at these loci (Fig. 5D).

Interferons play an important role in HSC biology, during development, at homeostasis, or during stress responses [62]. Both class I and II interferons directly signal to HSCs, and conflicting reports have emerged categorizing these cytokines as either promoting or suppressing hematopoiesis, depending on the experimental design. However, recent studies showed that treatment with IFNα [63] or IFNγ [64] induce cell cycle entry of quiescent HSCs. Of note, the overall HSC pool does not expand in these conditions due to impaired self-renewal [65]. While the relationship between cell cycle position and IFN signaling remains to be investigated, these observations could explain the impaired differentiation elicited by Sin3B inactivation in LT-HSCs.

A report in human HSCs demonstrated the transition from LT- to ST-HSCs was associated with cell cycle re-entry and changes in CTCF binding sites. These changes altered 3D chromatin interactions to repress stemness genes in ST-HSCs normally expressed in LT-HSCs [8]. Recent development of small molecule inhibitors of early cell cycle progression in clinical settings point to cell cycle progression as actionable opportunity for the modulation of hematopoietic stem and progenitor cells expansion/differentiation decision.

## Materials and methods

### Mice

Mice containing the Sin3B-flox (*Sin3B*^*F*^) allele have been previously described. To generate hematopoietic specific deletion of Sin3B, *Sin3B*^*F/F*^ mice were intercrossed to *Vav1-iCre* mice, which is active at embryonic day 11.5 (E11.5). *Ptprc*^*a*^*; Pepc*^*b*^ (CD45.1) congenic mice were purchased from The Jackson Laboratory and bred in-house to use as recipients for competitive transplantation experiments. All mice were kept on an inbred C57BL/6 background. Mice were housed in pathogen-free barrier facilities with a 12-h light/dark cycle and given food and water ad libitum. Mice were administered 5-Fluorouracil (Invivogen) via intraperitoneal injection at 100 mg/kg body weight. For competitive transplantation assays, recipient mice at 8–10 weeks of age were lethally irradiated (total body irradiation) with 12 Gy of *γ*-irradiation with a MultiRad 350 X-Ray Irradiator (Faxitron^®^). Mice were given 2 doses of 6 Gy of irradiation at least 3 h apart. Mice were maintained on sterile, acidified water supplemented with Sulfamethoxazole and Trimethoprim for 2 weeks following irradiation, replenishing the antibiotics after a week. Equal numbers of male and female mice were used in all experiments unless specified otherwise. All animal experiments and protocols were approved by the New York University Grossman School of Medicine Institutional Animal Care and Use Committee.

### Flow cytometry and cell sorting

To isolate indicated cell populations, mice were humanely sacrificed via CO_2_ inhalation and cervical dislocation was used as a secondary means of euthanasia. Femurs, tibiae, and pelvis were isolated from mice, and if increased numbers of cell were required, sternum, humeri, and vertebrae were dissected as well. Whole bone marrow was isolated from bones (femurs, tibiae, pelvis) through spinning in a microcentrifuge for 8 s into FACS-E buffer (1× phosphate buffered saline [PBS] supplemented with 2% fetal bovine serum [FBS] and 25 mM ethylenediaminetetraacetic acid [EDTA]) or through crushing in a mortar and pestle (sternum, humeri, vertebrae).

For whole bone marrow analysis, whole bone marrow was incubated in Ammonium-Chloride-Potassium (ACK) lysis buffer for 5 min on ice to remove erythrocytes. Cells were then resuspended in FACS buffer (1× PBS supplemented with 2% FBS) and incubated with a cocktail of biotinylated antibodies against lineage markers and Rat IgG (20 μg/mL) for 30 min on ice. Cells were washed and then incubated with HSPC antibodies conjugated to fluorophores and Rat IgG for 90 min. Cells are washed and resuspended in FACS buffer carrying 4′,6-diamidino-2-phenylindole (DAPI, 500 ng/mL) to mark dead cells and analyzed on either a Bectin, Dickinson, and Company (BD™) LSR II UV (equipped with 355 nm, 407 nm, 488 nm, 561 nm, 633 nm lasers) or a BD™ LSR II HTS (equipped with 407 nm, 488 nm, 561 nm, 633 nm lasers). Data collection was done using BD FACSDiva™ software and.fcs files were formally analyzed with FlowJo (FlowJo, BD). All flow cytometry experiments contained single color controls for compensation and gating.

For fluorescence activated cell sorting, whole bone marrow was first blocked with TruStain FcX™ PLUS (anti-mouse CD16/32) (50 μg/mL) for 5 min on ice in MACS Buffer (1× PBS supplemented with 1% FBS, 1% bovine serum albumin [BSA], and 2 mM EDTA that was sterile filtered through 0.22 μm filter and de-gassed). Then, cells were incubated with anti-mouse CD117 microbeads (20 μL for femurs, tibiae, and pelvis, 40 μL if also isolating cells from sternum, humeri, and vertebrae) for 15 min on ice. Cells were washed in MACS buffer and then filtered through a 40 μm mesh before being loaded onto a Miltenyi Biotec MS column placed in a miniMACS separator. Flowthrough containing CD117^−^ cells was discarded. MS column was washed with MACS buffer and then flowthrough discarded. Column was then removed from magnet and placed in a microcentrifuge tube. MACS buffer was loaded and plunger used to gently expel cells from the column. This CD117^+^ enriched fraction was then stained as previously described for lineage markers and HSPC markers before being resuspended in FACS buffer containing DAPI and passed through a 40 μm filter again and sorted on a BD FACSAria™ II (equipped with 355 nm, 407 nm, 488 nm, 561 nm, 633 nm lasers) or a BD FACSAria™ IIu SORP (equipped with 355 nm, 407 nm, 488 nm, 561 nm, 633 nm lasers) utilizing a 100 μm nozzle. Single color controls were used for compensation and florescence minus one controls were utilized to set gates before sorting using FACSDiva software. Cells were sorted into 1× PBS supplemented with 2% FBS. Analysis of sorting data was accomplished with FloJo software. Visualization and statistical analysis was computed after exporting data to GraphPad Prism9 software.

### Hematopoietic stem and progenitor immunophenotypes

The following markers were used for the indicated cell types: LT-HSC: L^-^S^+^K^+^Flk2^-^CD48^-^CD150^+^; ST-HSC: L^-^S^+^K^+^Flk2^-^CD48^-^CD150^-^; MPP2: L^-^S^+^K^+^Flk2^-^CD48^+^CD150^+^; MPP3: L^-^S^+^K^+^Flk2^-^CD48^+^CD150^-^; MPP4: L^-^S^+^K^+^Flk2^+^CD48^+^CD150^-^; L (Lineage) markers: CD3, CD4, CD8a, CD11b, B220, Gr1, IL7Rα, Ter-119; S: Sca-1 (Ly6a); K: c-Kit (CD117); Flk2: Flt-3 (CD135).  

### Competitive transplantation assay

Recipient CD45.1 mice at 8–10 weeks of age were irradiated the day before transplantation experiments, with split doses of irradiation at least 3 h apart. Donor *Sin3B*^*F/F*^ or *Sin3B*^*H−/−*^ were used at 6–8 weeks of age. Whole bone marrow was isolated erythrocytes lysed as described in Flow Cytometry and analysis section. Whole bone marrow cells were counted manually using a hemacytometer. 1 × 10^6^ donor wild-type or *Sin3B*^*−/−*^ cells were mixed at a 1:1 ratio with wild-type competitor CD45.1. Cells were washed with 1× PBS to remove traces of serum and 2 × 10^6^ cells were resuspended in 100μL sterile 0.22 μm filtered 1× PBS and transplanted into mice via retroorbital injection using a 31G, 6 mm insulin syringe (BD). After 8 weeks, mice were sacrificed and whole bone marrow was isolated and stained as described above for HSPC markers with the addition of antibodies to distinguish between CD45.1 and CD45.2 alleles. Data was analyzed using FloJo and statistically analyzed in Prism9.

### EdU incorporation assay

LT-HSCs were sorted as described above, and cultured in 96 well round bottom plates containing 100 μL of HSPC media (5% FBS, Stem Cell Factor [SCF, 25 ng/mL], Interleukin-11 [IL-11, 25 ng/mL], FMS-like tyrosine kinase 3 ligand [Flt-3L, 25 ng/mL], Thrombopoietin [TPO, 25 ng/mL], Interleukin-3 [IL-3, 10 ng/mL], Granulocyte–macrophage colony-stimulating factor [GM-CSF, 10 ng/mL], Erythropoietin [EPO, 4 Units/mL], 1% penicillin G/streptomycin, 2% GlutaMAXTM, 55 μM 2-mercaptoethanol in Iscove’s Modified Dulbecco’s Medium [IMDM]) for indicated time periods in a 37 °C humidified water- jacketed cell culture incubator with 5% CO_2_. At timepoints, cells were given 100 μL of fresh HSPC media containing 20 μM 5-Ethynyl-2′-deoxyuridine (EdU) for a final concentration of 10 μM. Cells were incubated for one hour, and then plated onto poly-L-lysine coated #1.5 coverslips placed in individual wells of 12 well plates. Cells were allowed to attach for 15 min at room temperature (RT). Then 800μL of BD Cytofix™ buffer was added to fix cells for 10 min at RT with gentle agitation. Then 200 μL of 1 M Glycine in ddH_2_O was added to quench fixation. Cells were washed three times with 1× PBS before proceeding to EdU staining.

Cells were processed with the Click-iT^®^ Plus EdU Imaging Kit. After washing, cells were permeabilized with Triton X-100 Buffer (0.5%[v/v] Triton X-100; 20 mM HEPES-KOH, pH 7.9; 50 mM NaCl; 3 mM MgCl_2_; 300 mM sucrose; 0.05% [w/v] NaN_3_ in ddH2O) for 10 min at RT with gentle agitation. Cells were washed twice with IF Washing Buffer (1% FBS; 1% BSA; 0.1% Triton X-100; 0.1%[v/v] Tween-20; 0.05% NaN_3_ in 1× PBS) before being incubated with Click-iT^®^ reaction cocktail containing Alexa Fluor^®^ 488 picolyl azide. Cells were incubated with reaction cocktail for 30 min at RT protected from light. Samples were then washed with IF Washing buffer containing 500 ng/mL DAPI, and then washed 3 more times with IF Washing buffer, and then once with 1× PBS, before being mounted on slides with Vectashield^®^ (Vector Labs). Slides were sealed with commercially available clear nail polish and allowed to dry before being imaging on an inverted Zeiss LSM 700 Laser Scanning Confocal Microscope (equipped with 405 nm, 488 nm, 555 nm, 639 lasers) using a 63× plan apochromat 1.4 oil objective. Zen software was used to acquire images, using 3.0 zoom and preventing saturation of images. Images were exported to FIJI (Fiji is just ImageJ) to quantify proportion of cells staining positively for EdU. Quantification was exported to Prism9 for additional analysis and visualization.

### Immunofluorescence of LT-HSCs

LT-HSCs from *Sin3B*^*F/F*^ and *Sin3B*^*H−/−*^ mice were isolated via FACS and plated on poly-l-lysine coverslips as described above for EdU labeling. After permeabilization, cells were washed twice with 1× PBS, and then blocked with 1× PBS supplemented with 5% (v/v) goat serum and 0.1% (v/v) Tween-20 for one hour at RT with gentle agitation. Coverslips were then flipped onto 100 μL droplets of blocking buffer (10% FBS, 2.5% BSA, 0.1% Tween-20, 0.1% Triton X-100, 0.05% NaN_3_ in 1× PBS) containing primary antibody at a 1:100 dilution. Cells were incubated with primary antibody for 1 h at RT. Coverslips were then returned to individual wells of 12 well plate and washed 3 times with IF washing buffer. Coverslips were flipped again onto droplets of blocking buffer containing secondary antibody. Goat anti-rabbit Alexa Fluor 488 or Goat anti-mouse Alexa Fluor 594 antibodies (Invitrogen) at a 1:400 dilution were used and cells were incubated for 1 h at RT protected from light. Cells were then washed 3 times in IF buffer, with the first wash carrying DAPI (500 ng/mL). Coverslips were washed once with 1× PBS before being mounted on slides in Vectashield. Samples were sealed with nail polish and imaged using a Zeiss 700 as detailed above. Z-stacks were taken and the widest slice of each cell was utilized for quantification using FIJI. A mask was manually drawn using DAPI to quantify the signal for the indicated antibody within the nucleus. A small area not containing cells was quantified to determine background. Corrected fluorescence was determined by the following formula: CorrFluor = Sample – (Area × Background). All data was exported to Prism9 for statistical analysis.

### Single cell RNA-sequencing: cDNA library preparation and sequencing

For homeostasis dataset, whole bone marrow was isolated as described above, and antibodies against Lineage, Sca1, and cKit were for 2 *Sin3B*^*F/F*^ and 2 Sin3B^H−/−^ male mice. LSKs were pooled and cells were blocked again with TruStain FcX PLUS for 5 min before wild-type cells were incubated with TotalSeq™-B0096 anti-mouse CD45 antibody and knockout cells with TotalSeq™-B0157 anti-mouse CD45.2 antibody for 15 min. Cells were washed with FACS buffer before being manually counted with a hemacytometer. The 10× Genomics Chromium Single Cell 3′ v3 kit was used to generate single cell suspensions. After counting, 2.5 × 10^4^ cells from each genotype were combined with MasterMix and loaded onto a Chromium Chip along with the gel beads and partitioning oil. Gasket was carefully placed over chip and was loaded into a Single Cell Controller. GEMs were carefully pipetted out and visually inspected before being placed in a thermal cycler for cDNA synthesis with the following parameters (Step 1—53 °C for 45 min; Step 2—85 °C for 5 min; Step 3—4 °C: Hold).

Reaction was stored at − 20 °C until cDNA library preparation. Quality of cDNA was checked with an Agilent 2100 Bioanalyzer. Libraries were generated via using the 10× Genomics 3′ GEM protocol with HTO primers and sequenced on an Illumina NovaSeq.

For dataset of LSKs at stress, the same workflow was utilized, except mice were first given a single dose of 5-fluorauracil (100 mg/kg) injected intraperitoneally. After 9 days, mice were sacrificed and bone marrow was processed and stained as previously described and LSKs were isolated via FACS utilizing the same strategy.

### Single cell RNA-Seq: analysis

After sequencing, reads from cDNA and Hashtag oligos (HTOs) were demultiplexed and aligned using the 10× Genomics CellRanger software. This generated a matrix file, features file, and barcodes file that was imported into R. The Seurat package was used for downstream analysis. Briefly, the output files from CellRanger were used to generate a Seurat object. HTO counts were extracted and added to the metadata of captured cells. Data was normalized and the HTODemux function was used to classify cells. Only singlets were kept for downstream analysis. Quality control was used to filter to calculate the distribution of genes per cell identified, as well as overall counts and the proportion of reads coming from the mitochondria. Cells in the top and bottom 2% of these metrics were filtered out. Cells found to be expressing any lineage markers used in the FACS isolation were also removed. In addition, cells expressing genes related to biased and lineage-primed HSCs were also removed.

Next, LSKs were assigned a subset identify utilizing previously published transcriptional signatures of LT-HSCs, ST-HSCs, MPP2s, MPP3s, and MPP4s. To generate Uniform Manifold Approximation Projections (UMAPs) data was scaled and Principal Component Analysis (PCA) was computed. JackStraw was then used to calculate statistically significant PC’s to use for UMAP analysis. Differentially expressed genes were determined using the FindMarkers function. The dataset containing LSKs at stress was analyzed using the same workflow as just described.

To integrate the homeostasis and stress datasets, we utilized Seurat’s IntegrateData function. First, variable features were calculated for both datasets, and integration anchors were calculated using FindIntegrationAnchors. These anchors were then used for the IntegrateData function to generate the object containing cells from homeostasis and stress.

For pseudotime analysis, the Monocle3 package in R was used. First, the relevant Seurat object was converted into a CellDataSet format using the as, cell_data_set function from the SeuratWrappers package. The UMAP calculation and LSK subsets defined in Seurat were used in the learn_graph function of Monocle3 when determining cell trajectories. The order_cells function was used to determine pseudotime, with the node containing the most LT-HSCs manually selected as the beginning of the pseudotime trajectory. The graph autocorrelation analysis was completed using the graph_test function using the “principal_graph” that constituted the previously calculated trajectory in learn_graph and order_cells. Genes with a q_value < 0.05 were selected and modules of co-regulated genes as a function of pseudotime were determined via find_gene_modules. Aggregate expression of genes within individual modules was accomplished via the aggregate_gene_expression function and graphed. Modules were exported and gene lists uploaded to Enrichr to determine gene ontologies.

For cell cycle analysis, cell cycle scores utilizing previously published datasets were calculated using log10 transformed expression. LT-HSCs were ordered from M/G_1_, G_0_, G_0_/G_1_, G_1_/S, S, G_2_/M, and M. Cells were ordered from lowest to highest expression of their G_0_ score, and data were transformed into percentile ranks to normalize for cell number.

### Assay for transposase-accessible chromatin using sequencing (ATAC-Seq)

To conduct ATAC-Seq on LT-HSCs, we utilized the ATAC-Seq kit from ActiveMotif with the following modifications. LT-HSCs from 2 mice per genotype in duplicate were pooled after sorting via FACS as previously described. The amount of Assembled Transposomes was scaled based on the number of cells we were able to isolate. After tagmentation for 30 min, the rest of the kit was followed per manufacturer’s instructions. Briefly, DNA Purification Binding Buffer was added to samples and transferred to a DNA binding column. Columns were washed and DNA was eluted for subsequent PCR amplification. Illumina’s indexed i7 and i5 Nextera primers were used to distinguish between samples. DNA was amplified using Q5 polymerase and specific primer combinations for 10 cycles with the following conditions on a thermal cycler (Step 1—72 °C, 5 min; Step 2—98 °C, 30 s; Step 3—98 °C, 10 s; Step 4—63 °C, 30 s; Step 5—72 °C, 1 min; Repeat Steps 3–5 nine more times for a total of 10 cycles; Step 6–10 °C, hold). SPRI (Solid Phase Reversible Immobilization) beads were used for clean-up. Beads were washed twice with ethanol and DNA was eluted from beads. Size distribution of libraries was determined using a TapeStation, and concentration with Bioanalyzer.

Our samples required additional PCR cycles and the same process was repeated on libraries for an additional 2 cycles before bead clean-up was repeated. Libraries were sequenced on an Illumina NovaSeq. Fastq files were run through FastQC and trimmed. Reads were then aligned using bowtie2 and duplicates were removed using sambamba. Bigwig files were generated using deeptools and peaks were called using MACS2.

Bed files containing peaks were imported into R and the DiffBind package was used for downstream analysis. Peaksets were read in and normalized before differential analysis was calculated using DESeq2. The HOMER package was used to annotate peaks and to calculate enrichment of DNA-binding factor motifs. The bedtools suite was utilized to compare peaksets to each other, with the intersect function used to directly compare lists of accessible peaks. Peaks were then fed into the GREAT tool using default parameters to determine putative genes regulated by the accessible chromatin peaks we identified. Finally, those genes were then used as input for Enrichr to determine Gene Ontology enrichment. Individual ATAC-Seq tracks were loaded by opening bigwig files in IGV.

For motif analysis, the HOMER function findMotifsGenome.pl was used for individual peak lists. Primed peaks list were taken from Martin, et al. [52] and first changed to an mm10 annotation format using the UCSC genome browser tool. Lists were directly compared to ATAC-seq peaks using bedtools.

### Statistical considerations

Samples were compared using the statistical test indicated in figure legend.

Sample sizes were not determined with any formal power calculation.

## Supplementary Information

**Additional file 1: Table S1.** Cell cycle signatures used to classify phase as detailed in Fig. 4.

**Additional file 2: Figure S1.** The adult murine hematopoietic hierarchy. Depicted is a simplified view of the hematopoietic hierarchy of adult mice, with LT-HSCs residing at the top, which give rise to more lineage-restricted progenitors with limited self-renewal properties (MPPs). These MPPs then give rise to even more restricted oligopotent progenitors, that will produce unipotent progenitor cells that terminally differentiate into the effector cells shown.

**Additional file 3: Figure S2.** LT-HSCs express lineage-related transcription factors independent of Sin3B status. **a** Transcription factor expression for specific lineage in LT-HSCs from Sin3B^+/+^ or Sin3B^H−/−^ mice at homeostasis. No significant differences were found in listed transcription factors in LT-HSCs that were identified with supervised clustering. Expression from normalized counts shown. **b** Expression of the same transcription factors in (**a**). from Sin3B^+/+^ and Sin3B^−/−^ LT-HSCs from mice treated nine days prior with 5-FU. Only Meis1, an HSC stemness gene, was shown to be significantly upregulated in Sin3B^−/−^ LT-HSCs. Other differentiation-related transcription factors were normally expressed. Expression from normalized counts shown.

**Additional file 4: Figure S3.** Loss of Sin3B in LT-HSCs results in aberrant chromatin accessibility. ATAC-Seq was performed on FACS-purified LT-HSCs from Sin3B^+/+^ and Sin3B^H−/−^ mice. **a** Correlation analysis reveals that wild-type samples (WT1, WT2) cluster closely to one another, and are distinct from Sin3B^−/−^ samples (KO1, KO2). **b** Analysis of all peaks from samples shows an increase in the level of accessibility in Sin3B^−/−^ LT-HSCs. Each sample represents LT-HSCs pooled from two mice of the indicated genotype, for a total of 8 mice. Each sample is a biological replicate.

**Additional file 5: Figure S4.** Sin3B is required for promoter silencing. **a** HOMER annotations of all ATAC-Seq peaks recovered separated by genotype. **b** Annotations and proportions of peaks differentially accessible in Sin3B^+/+^ LT-HSCs. **c** Annotations and proportions of peaks differentially accessible in Sin3B^−/−^ LT-HSCs. Differential accessibility was determined to be statistically significant with a q value < 0.05.

**Additional file 6: Figure S5.** Correlation between RNA-Seq and ATAC-Seq data in LT-HSCS. Differentially regulated genes in Sin3B^−/−^ LT-HSCs compared to Sin3B^+/+^ LT-HSCs at homeostasis. Over 100 genes were determined to be significantly differentially expressed, and over 40 of those genes had ATAC-Seq peaks annotated to them. Plotted are the fold changes in ATAC-Seq peaks for genes compared to their RNA-Seq fold change.

## Data Availability

All sequencing data reported in this article have been deposited in the NCBI Gene Expression Omnibus database GEO: GSE301007 and GSE301084. Other data or protocols are available upon request from the corresponding author Gregory David (Gregory.David@nyulangone.org).
